# Enhanced Healing and Antimicrobial Efficacy of Chitosan-g-Polyacrylamide in a Rat Model of Gingival Ulcers

**DOI:** 10.3389/fchem.2020.00273

**Published:** 2020-04-24

**Authors:** Yanfen Zheng, Lingjie Ke, Yin Lu, Qiliang Zuo, Guanhong Deng, Hairui Wang, Xiamei Zeng

**Affiliations:** ^1^Department of Oral Mucosal Diseases and Department of Prosthodontics, Stomatological Hospital of Xiamen Medical College, Xiamen, China; ^2^Fujian Provincial Key Laboratory of Innovative Drug Target Research and State Key Laboratory of Cellular Stress Biology, School of Pharmaceutical Sciences, Xiamen University, Xiamen, China

**Keywords:** oral, ulcerative lesion, anti-bacterial, chitosan, polyacrylamide

## Abstract

Patients in dental hospitals often experience oral ulcerative lesions, which lead to pain and affect the patient's quality of life. At present, the goal of treating oral ulcerative lesions with drugs is to reduce inflammation and promote ulcer healing. However, very few antibacterial and hemostatic drugs are designed to be suitable for the microenvironment of gingival ulcers. Based on this, we have designed a natural therapeutic agent for oral ulcerative lesions that meets the various requirements of oral ulcerative lesion medication. The chitosan-g-polyacrylamide (CP) copolymer is composed of chitosan as the main chain and polyacrylamide polymers as the side chains. Antibacterial experiments show that this polymer can effectively inhibit the proliferation of Gram-negative (*Escherichia coli*) and Gram-positive bacteria (*Staphylococcus aureus*). *In vitro* cell experiments also show that the CP copolymer is non-toxic, which is conducive to ulcer wound healing. Coagulation experiments prove that the CP copolymer can accelerate blood coagulation to stop bleeding. In experiments using a Wistar rat gingival ulcer model, the CP copolymer significantly promoted ulcer healing and shortened the healing time. These results indicate that the CP copolymer may serve as a potential therapeutic agent for oral ulcerative lesions.

## Introduction

Oral ulcerative lesions result in a loss of epithelial tissue in the oral mucosa membrane and are often seen in patients in dental hospitals (Trautwein-Weidner et al., 2015). Recurrent aphthous ulcers are considered to be the most common oral mucosa disease, with an incidence rate ranging from 5 to 60% in the general population (Warinner et al., 2014). Radiation stomatitis is a radiation-induced disease that generally occurs in patients who have received radiotherapy for head or neck cancer. Severe radiation stomatitis can be seen in 29 to 66% of all head and neck cancer patients after undergoing radiotherapy (Sumita et al., 2014). The extremely painful symptoms associated with these ulcerative diseases may lead to difficulty in chewing, swallowing, and speaking. Such functional limitations may negatively influence the patient's quality of life (Lee et al., 2016). They are often accompanied by halitosis, nausea, chronic pharyngitis, lymph node swelling, and other complications (McGuire et al., 2013).

Treatments for chronic oral wounds require effective adherence to the wound surface and the ability to prevent infection and promote wound healing. Thus, the therapeutic materials used for this purpose must display appropriate mechanical and swelling properties. At present, oral ulcerative lesion treatments are primarily developed to relieve pain, reduce inflammation, promote ulcer healing, and prolong the attack interval (Vieira et al., 2012) In addition to basic oral care, medications for oral ulcerative lesions include growth factors, anti-inflammatories, cytokines, glucocorticoid, and adrenal hormones (Raber-Durlacher et al., 2013). However, the efficacy of therapeutic drugs may be affected by muscle movement and the continuous secretion of oral saliva, resulting in a low effective concentration of the drugs. With the continuous progress that is being made in mucous membrane adhesives, drug delivery carriers, permeability enhancers, and other technologies, the ability to implement safe and effective drug delivery to treat local or systemic diseases has increased. The progress made in oral biomaterial research has the potential to improve the therapeutic effect of oral ulcerative lesion drugs and decrease drug waste (Martín et al., 2015). Silver nanoparticles, which are natural materials, have been used in wound dressings to defend against local wound infections. However, silver is a strong preservative, and its inevitable toxicity will cause varying degrees of damage to the human body (Wilkinson et al., 2011; Karim et al., 2016). Thus, the development of a biocompatible drug delivery carrier is clearly needed for ulcerative lesions.

Chitosan (CS) is a N-deacetylated derivative of chitin that can be effectively degraded into non-toxic, absorbable monosaccharides that are not teratogenic (Bonferoni et al., 2009; Elsabee et al., 2009). It is the second-most naturally abundant biodegradable polymer material and is used in the biomedicine industry due to its antibacterial, anti-inflammatory, anti-thrombotic, hemostatic, wound healing, and immunomodulating effects (Wang and Wang, 2011; Lewandowska, 2015). In recent years, chitosan-grafted polymers have been used in cell stents and wound healing polymer dressings in the biomedicine field (Nettles and Elder, 2002; Berger et al., 2004; Kalambettu and Rajangam, 2012). The surface affinity structure of the double electron layer formed by chitosan contact with mucosal epithelial cells, along with its swelling properties, aids in its adhesion in the oral environment. Chitosan water absorption relaxes the polymer chain from a crimped to a flat state, which increases structural exposure to further increase adhesion.

Although it has many advantages, its poor water solubility significantly limits its use in biological applications. However, the water solubility and biological activity of chitosan can be improved through chemical modification. Mahattanadul et al. found that a 0.1% chitosan turmeric mouthwash can be used as a potential local therapeutic agent for oral inflammation-type ulceration and anti-candidiasis (Mahattanadul et al., 2018). Zhang et al. constructed a Matrine/CS bio-film with biodegradable chitosan as the carrier material, which displayed good antibacterial performance, biocompatibility, and anti-inflammatory properties during healing of the aphtha (Zhang C. et al., 2019). The clinical trial results of Shao et al. showed that chitosan can effectively promote drug penetration, robustly adheres to oral mucosa epithelial cells, has a strong curative effect, and can be applied as an excellent medicine for treating oral ulcerative lesions (Shao and Zhou, 2019).

Polyacrylamide (PAM) is a hydrophilic, synthetic anion/non-ionic polymer with an active amino group along the main chain. Recent studies have shown that the scaffold structure formed by PAM polymers can effectively promote the proliferation of fibroblasts with a low level of cytotoxicity (Risbud and Bhonde, 2000; Zhang et al., 2005; Lewandowska, 2007). Grafting PAM onto the chitosan backbone increases polymer stability, promotes fibroblast proliferation, and accelerates ulcer healing, making chitosan a viable material for therapeutic applications (Feller et al., 2010). Some studies have shown that the presence of a carbodiimide moiety can mediate the formation of an amine bond between PAM and another polymer, such as chitosan, to form a copolymer conjugate system. In addition, free-radical polymerization can be used to graft PAM onto chitosan using glutaraldehyde or tetraethylene acrylate as a cross-linking agent (Kandow et al., 2007;Sheng et al., 2014).

In this study, we grafted polyacrylamide to chitosan [chitosan-g-polyacrylamide (CP) copolymer] to increase its solubility for the treatment of oral ulcerative lesions. The CP copolymer was fabricated by conjugating chitosan with several polyacrylamide side chains. The ^1^H NMR and FTIR spectra indicated that polyacrylamide was effectively grafted onto the main chain of chitosan by *in situ* free-radical polymerization. We further determined its hemostatic effect, antibacterial activity, and its effect on gingival fibroblast proliferation. An oral gingival ulcer mucous membrane model established using Wistar rats was used to investigate the therapeutic effect of the CP copolymer system. Results showed that the CP copolymer system effectively promoted coagulation, bacterial resistance, and promoted gingival fibroblast proliferation. These findings have not previously been reported and suggest that chitosan is a promising therapeutic agent for oral ulcerative lesions.

## Experimental Methods

### Chemical Materials

Acrylamide (AM, >98%), ammonium persulfate (APS, >98%), acetic acid (>99.5%), and ethanol were purchased from Sigma-Aldrich Co., Ltd. (Canada). *N,N,N*′*,N*′-tetramethyl- 1,2-ethanediamine (TEMED, >98%), Chitosan (CS, deacetylation value, 75–85%), and sodium hydrate (NaOH) were obtained from TCI (Tokyo Chemical Industry Co., Ltd, Japan). Tryptone and yeast extract were obtained from OXOID Co., Ltd (USA). Agar Powder was purchased from Solarbio Co., Ltd (China). Sodium chloride (NaCl, 99.9%) was obtained from JHD Co., Ltd (China). DMEM/F-12 culture medium, trypsin-EDTA (0.25%), penicillin-streptomycin, cell counting kit-8 (CCK-8), and the hematoxylin and eosin (H&E) staining kit were all obtained from Yeasen Biotech Co., Ltd (China). Fetal bovine serum (FBS, Gibco) was purchased from Thermo Fisher Co., Ltd (USA). Chloral hydrate, D_2_O, and DCl was obtained from Macklin Biochemical Co., Ltd (China).

### Synthesis Route of CP Copolymer

Based on our recently reported work with slight modifications, the chitosan-based polymer was synthesized using an *in-situ* polymerization method in dilute acetic acid (Zhang C. et al., 2019). Chitosan (0.4 g) was dispersed in a dilute acetic acid aqueous solution (1%, 20 mL) and stirred violently at room temperature for 4 h. The insoluble substances were removed by filtration. Then the AM monomer (AM:CS = 7:2 mass ratio) was added to the reaction solution and continuously stirred under N_2_ for ~20 min at room temperature. APS and TEMED catalysts (1:1 molar ratio at 3 mol% of the monomer concentration) were then added to the solution and stirred for 10 min. Subsequently, the mixture was heated to 30°C for 24 h. The solution pH was adjusted to 9.0 with a NaOH solution (1 M) to obtain the precipitant. The addition of a few milliliters of ethanol further facilitated precipitation. This purification process was repeated twice. After that, the system was dialyzed for 3 days at room temperature to eliminate unreacted substances (spectrum dialysis membrane, MWCO 10 kDa). This solution was then frozen and dried to obtain a light-yellow solid. The yield of the CP copolymer was ~85%.

### Characterizations of the CP Copolymer by FTIR and 1H NMR

FTIR spectra were recorded using a KBr pellet (4,000–400 cm^−1^) on a Perkin-Elmer FTIR2000 spectrometer (Perkin-Elmer Co., Ltd, UK). An average of 64 scans were taken at room temperature with a resolution of up to 2 cm^−1^. The coupling sample was dried in a vacuum at 50°C for 1 day before testing. ^1^H NMR spectra of conjugates at room temperature were measured with a JEOL ECA 500 MHz NMR spectrometer (JEOL Co., Ltd, Japan). D_2_O, containing 0.1 M DCl, was used as the solvent. The chemical shifts were reported in 1/1,000,000 (ppm). The solvent peak of D_2_O is δH 4.70.

### Antibacterial Behavior of the CP Co polymer

The antibacterial effects of the CP copolymer were investigated using an inhibition zone method (Lu et al., 2017). *Staphylococcus aureus* (1,000,000 CFU/mL) and *Escherichia coli* (1,000,000 CFU/mL) cultures were evenly coated on a solid LB medium plates. Circular copolymer samples (with a diameter of 6 mm) were then deposited on the surface of the solid medium; the plates were then incubated at 37°C for 24 h. The antibacterial effect of the CP copolymer at different concentrations was evaluated by observing the size of the inhibition zones around the hydrogel samples.

### Cell Culture

Human gingival fibroblasts (HGF-1, Shanghai Sixin Biotech.Co., Ltd., China) were cultured in a cell incubator with DMEM/F12 media, FBS (10%), and penicillin-streptomycin (100 μg/mL) with 5% CO_2_ at 37°C.

### Cell Viability Assay

The viability of HGF-1 cells incubated with the CP copolymer was investigated using a CCK-8 assay. Cells were cultured in sterile 96-well plates at 4.8 × 10^5^ cells/mL (4.8 × 10^4^ cells per hole) with different concentrations of the CP copolymer for 24 h. Next, 10 μL volume of CCK-8 reagent was added to each well, and sterile 96-well plates were incubated for another 3 h. The absorbance of the samples at a wavelength of 450 nm was measured using an enzyme labeling instrument (Perkin Elmer Co., Ltd, USA).

### Cell Growth Evaluation

The cell growth evaluation of HGF-1 cells was determined using the CCK-8. When the number of HGF-1 cells reached 2.88 × 10^5^ cells/mL, the cells were cultured in sterile 96-well plates with different concentrations of the CP copolymer for 24 h. Fresh media was replaced every day. Next, 960 μL of CCK-8 was added to the 96-well plates at 12, 24, and 48 h of culture and sterile 96-well plates were incubated for 4 h. The absorbance wavelength (450 nm) was measured with an enzyme labeling instrument, and cell growth evaluation were calculated for each time point from the average OD values.

### Establishment of Animal Models

Filter paper (3 mm × 3 mm) samples with 20 μL of a 70% acetic acid solution were applied (for 1 min) to the gingival mucosa location of healthy mature Wistar rats (Shanghai SLAC Laboratory Animal Co.,Ltd, China) that were anesthetized with 10% chloral hydrate. Application of 70% acetic acid was repeated for two consecutive days to establish the animal model. Animal treatment procedures and feeding were conducted as per the Xiamen University Animal Care Guidelines.

### Analysis of the Hemostatic Effect of the CP Copolymer

The blood of Wistar rats was collected by retro-orbital extraction into EP tubes that contained heparin. The blood was mixed with the CP copolymer samples (2:1 volume ratio) and incubated in a water bath at 37°C. Untreated rat blood was used as the control group.

### *In vivo* Effects of the CP Copolymer on Gingival Mucosal Ulcers

After animal models were established, 20 μL of a 5 wt% CP copolymer solution was applied to the festering site in the oral cavity of anesthetized rats [chloral hydrate (10%)] every day. Each experimental group contained three rats, and pictures were taken every 5 days with a camera to measure and record the length and width of the oral cavity using Vernier calipers.

### Tissue Section Analyses

After 10 days of administration, the rats were sacrificed, and tissues were taken isolated and immersed in 15 or 30% sucrose solutions for 24 h. The tissues were frozen and sliced into sections of 7 μm in thickness, then stained with an H&E stain for microscopic observation. All animal treatment procedures and feeding were conducted following the Xiamen University Animal Care Guidelines.

### Statistical Analyses

All experimental data are represented as the mean ± standard deviation. All experimental data and figures were analyzed using GraphPad Prism 5.0 and OriginPro 8. Statistical significance was indicated when the *p*-value was <0.05.

## Results and Discussion

### Synthesis and Characterization of the CP Copolymer

**Figure 1:**
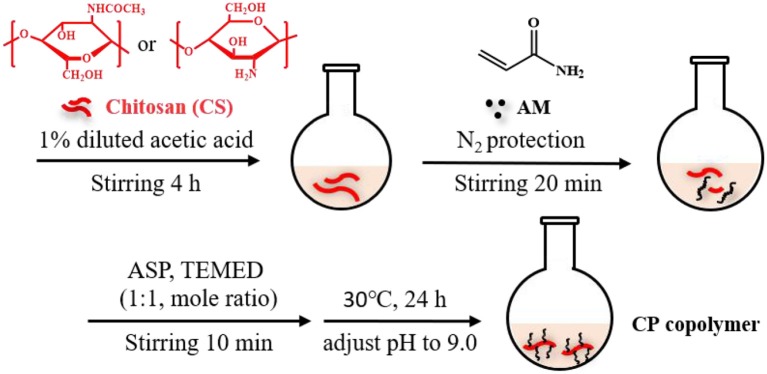
Synthetic scheme of chitosan-based CP copolymer formation by *in situ* free radical polymerization.

Our recent work presented a simple, one-pot procedure to prepare chitosan conjugate compounds (Zhang C. et al., 2019; Zhang Z. et al., 2019). The CP copolymer was prepared by *in-situ* free-radical-induced polymerization using TEMED as an accelerator and APS as an initiator in an acetic acid solution (Figure 1). The sulfate radical produced by thermal decomposition of the APS initiator caused AM polymerization to create a PAM long chain structure, which further polymerized with chitosan *in situ* (Huang et al., 2013; Echeverria et al., 2015; Wang and Yu, 2015). CP copolymers were synthesized with a mass ratio of 3.41:1 (PAM:CS). The characteristic chemical shift peaks of PAM and chitosan could both be observed in the ^1^H NMR spectra of the CP copolymer (Figure S1). The specific characteristic peaks were as follows: 2.38–1.10 m (protons of the main chain, br); 0.97 m [-CH(C*H*_3_)_2_, br] belong to the PAM polymer; 4.12–2.77 m (protons, br), 2.28–1.98 m [-CH_2_-C*H*(CONH_2_)-, br], 1.93–1.88 m (-NHCO-C*H*_3_, br), and 1.79–1.21 m [-C*H*_2_-CH(CONH_2_)-, br] belonging to chitosan. The mass ratio (calculated by signal integration from the ^1^H NMR spectra) of PAM and chitosan in the conjugates was close to that of the initial ratio of monomer to chitosan. The monomer conversion rate of PAM was typically higher than 99%, and the conjugation yield was higher than 85%. The above data indicated that the polymer was grafted onto the chitosan backbone.

The FTIR spectra of conjugates and chitosan were collected to determine the extent of PAM grafting onto the chitosan backbone (Figure S2). The characteristic peaks of chitosan were seen at 1,081 cm^−1^ [s, ν(C-O-C) and ν(C-O)], 1,650 cm^−1^ [s, ν(C=O)], 2,879 cm^−1^ [m, ν_s_(C-H) and ν_as_(C-H)], and 3,440 cm^−1^ [strong and wide, br, ν(O-H) and ν(N-H)]. The FTIR spectrum of the CP copolymer also showed additional peaks at 1,452 cm^−1^ [m, ν(C-N)] due to the amide group of PAM, 1,658 cm^−1^ [s, ν(C=O), 2,930 cm^−1^ (m, ν_as_(C-H)], and 3,000–3,750 cm^−1^ [br, ν(O-H) and ν(N-H)] due to the amide groups of chitosan and PAM. These FTIR results further indicated that PAM grafting onto the chitosan backbone was achieved by *in situ* free-radical polymerization.

### Antibacterial Effects of the CP Copolymer

**Figure 2:**
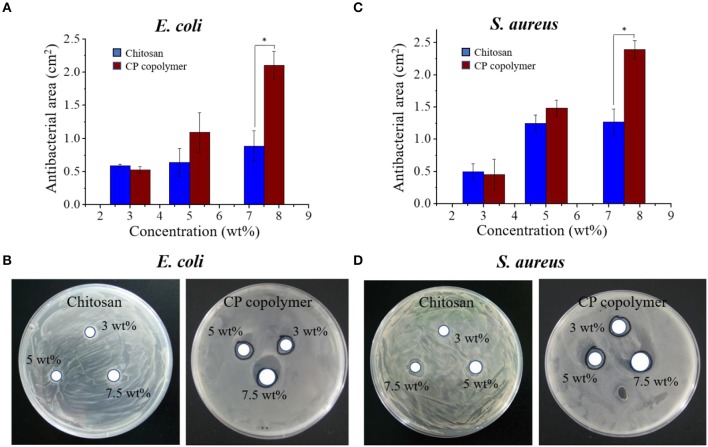
**(A)** The antibacterial activity of the CP copolymer and chitosan against *E. coli* as determined by the antibacterial area during culture (24 h, 37°C). **(B)** The antibacterial activity of the CP copolymer and chitosan against *S. aureus* as determined by the antibacterial area during culture (24 h, 37°C). **(C)** The quantitative data analysis for **(A)**. **(D)** The quantitative data analysis for **(B)**. *p < 0.05.

Oral bacterial infections often increase the burden of ulcer wound healing, necessitating effective antibacterial treatments. While the antibacterial activity of chitosan is well-known (Sonis et al., 2014; Zivanovic et al., 2007), its specific antibacterial mechanism has not been definitively established. Dina et al. suggested that chitosan's antibacterial ability is regulated by a number of diatheses, which are concentration-dependent and may be related to binding of the tea polyacid and potential extraction of lipids (Xiao et al., 2000; Dina et al., 2008). Other research indicates that the pH, temperature, and the ionic strength in the microenvironment strongly influences chitosan's antibacterial ability. At an acidic pH (pH < 6) and high temperature (25–37°C), the increase in ionic strength can significantly improve the sterilizing ability of chitosan, but the metal ions can inhibit the antibacterial effects of chitosan (Guo-Jane and Prot, 1999; Chung et al., 2003). In this study, the antibacterial activity of the CP copolymer system was investigated using *E. coli* and *S. aureus* (Figure 2). The CP copolymers were transferred to the *E. coli* and *S. aureus* mediums, and the growth status of the colonies near the samples was observed. Bacterial cultures were challenged with three CP copolymer concentration gradients (3.0 to 7.5 wt%) to verify the relationship between sample concentration and antimicrobial activity. It was easy to observe that the CP copolymer showed antibacterial activity as the antibacterial area (cm^2^) increased with the increasing polymer concentration. The high antibacterial activity of the CP copolymer is likely related to the high specific gravity of chitosan.

### CP Copolymer Mediates *in vitro* Human Gingival Fibroblast Proliferation

**Figure 3:**
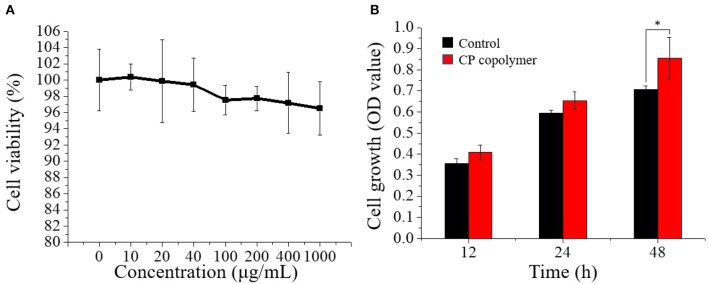
**(A)** Cell viability of human gingival fibroblast cells treated with the CP copolymer (0–1,000 μg/mL). **(B)** Cell growth (OD_450_) following the treatment of human gingival fibroblasts with the CP copolymer.*p < 0.05.

The ability of the CP copolymer to mediate human fibroblast proliferation in the oral cavity was investigated with human gingival fibroblasts (HGF-1), which are often used to study mucosa-related diseases (Häkkinen et al., 2014; Vahabi et al., 2015). The CP copolymer (0 to 1,000 μg/mL) demonstrated no cytotoxic effects to the HGF-1 cells during 48 h of culture (Figure 3A). A CCK-8 assay was used to determine the effects of the CP copolymer on HGF-1 cell growth (Figure 3B). Surprisingly, the CP copolymer strongly promoted HGF-1 cell growth. Clearly, the CP copolymer system significantly promotes cell DNA replication, thereby promoting cell proliferation. The specific gravity of chitosan and PAM caused the increase in HGF-1 cell proliferation in this system.

### Hemostatic Effects of the CP Copolymer

**Figure 4:**
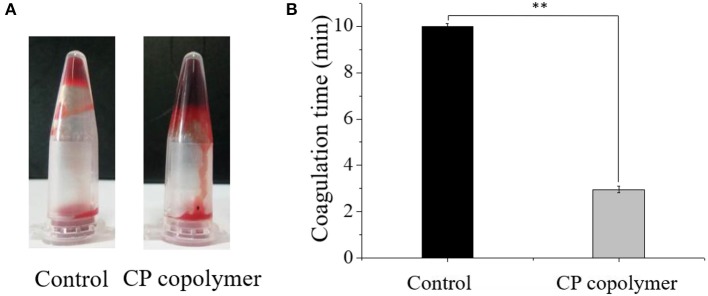
**(A)** Pictures of blood incubated with the CP copolymer (5 wt%) in a microcentrifuge tube after 30 s of treatment in a water bath at 37°C. **(B)** Coagulation time was quantified, for which samples from the control group were assigned the maximum clotting time for the assay as they did not clot. ** *p* < 0.01.

Oral ulcerative lesions are often accompanied by bleeding, which causes patient discomfort and is not conducive to wound healing. Therefore, a hemostatic polymer system is needed to treat oral ulcerative lesion diseases (Lee et al., 2014). Natural, biodegradable chitosan is the only positively charged cationic polysaccharide that can interact with negatively charged red blood cells to form a thrombus (Murugan and Ramakrishna, 2006; Yang et al., 2008). Chitosan can also activate platelets and inhibit the dissolution of fibrin, thereby promoting blood coagulation and hemostasis (Wu et al., 2004; Zigang et al., 2004; Li et al., 2016). To investigate the hemostatic effects of the CP copolymer, blood was collected from Wistar rats (in heparinized EP tubes) by retro-orbital blood sampling. The blood was then mixed with the CP copolymer and incubated in a water bath at 37°C. As can be seen from (Figure 4A), the CP copolymer clotted the blood after 30 s. (Figure 4B) shows that the coagulation time of the CP copolymer was much shorter than that of the control due to the combination of the chitosan skeleton and PAM.

### *In vivo* Therapeutic Effect of the CP Copolymer on Rat Gingival Mucosal Ulcers

**Figure 5:**
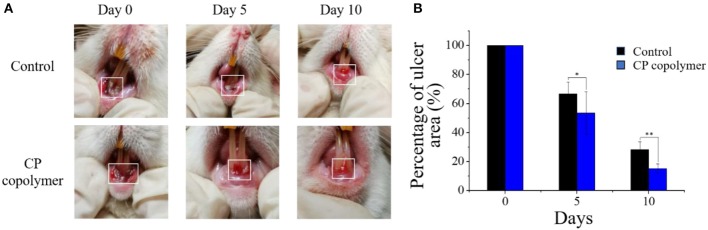
*In vivo* evaluation of the healing effects of the CP copolymer on oral gingival mucosa ulcers. **(A)** Photographs of the oral gingival swelling model in Wistar rats treated with the CP copolymer. **(B)** Gingival swelling areas were measured after dropping normal saline and the CP copolymer onto the ulcerated area. *p < 0.05,**p < 0.01.

**Figure 6:**
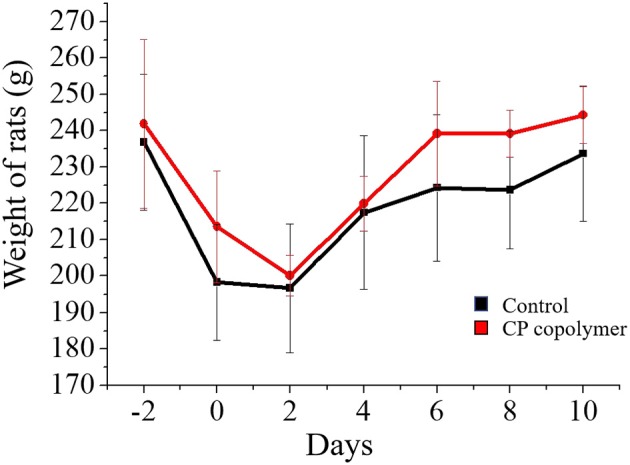
Weight curve of Wistar rats treated with CP copolymer compared to the case for PBS treatment (in the control group).

As the *in vitro* experiments showed that the CP copolymer had a promoting effect on the proliferation of human gingival fibroblasts, the *in vivo* effects of the CP copolymer on oral ulcers were investigated to determine its potential as a therapeutic agent. An oral ulcerative lesion model in Wistar rats was used to investigate the therapeutic effect of the CP copolymer by monitoring the degree of healing every 5 days over a 10-day period (Figure 5A). The swelling area of rats treated with the CP copolymer after 10 days was much lower than the swelling area of rats in the control group.

(Figure 5B) summarizes the change in the gingival ulcer area over 10 days, which showed that the CP copolymer had a reduction of 46% in the ulcer area within 5 days, compared to a 33% reduction in the area in the control group. It is worth mentioning that after 10 days of treatment, the ulcer area in the CP copolymer group decreased by 85%, compared to a 72% decrease in the control group, indicating that the CP copolymer effectively promoted the healing of oral ulcerative lesions. (Figure 6) shows the change in body weight of the Wistar rats, which was used to further determine the treatment effect on oral ulcerative lesions. It can be seen that the body weight of the five control group rats showed an acute downward trend in the first two days, which may be caused by difficulties in eating due to the induced oral ulcerative lesion. The body weights of rats that recovered after treatment with the CP copolymer were higher than those of rats in the control group, indicating that the CP copolymer treatment resulted in a gradual reduction of the oral ulceration symptoms and the recovery of feeding. This indicates that the CP copolymer effectively improved oral festering and promoted wound healing in Wistar rats. The CP copolymer displayed antibacterial effects and simultaneously promoted gingival fibroblast proliferation, making it an effective gingival ulcer treatment.

### Histological Sections of Gingival Mucosal Ulcers Treated With the CP Copolymer

**Figure 7:**
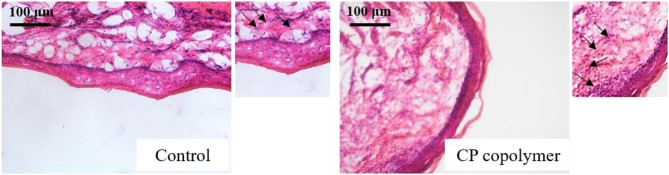
Hematoxylin & eosin staining of excised oral gingival ulcer tissue sections from Wistar rats after treatment with the control (PBS) or CP copolymer for 10 days. (The black arrows represent fibroblasts).

To further investigate the therapeutic effects of the CP copolymer on rat oral gingival mucosa ulcers, histological sections of gingival ulcer sites were analyzed (Figure 7). The gingival ulcer tissue sections were stained with H&E stain, and then observed under a light microscope. Purple-blue staining (hematoxylin) mainly shows chromatin and nucleic acids, and purple-red staining (eosin) shows the extracellular matrix and cytoplasm. In the untreated control group, the cells between the basal layer and the keratinized layer of the epidermis were relatively loose with clear inflammatory cell infiltration. Compared with the control group, the number of newly formed fibroblasts in the CP copolymer group was significantly increased, and the tissue damage was decreased.

## Conclusions

The biodegradable CP copolymer was successfully synthesized by *in situ* free-radical polymerization and consisted of a chitosan main chain and PAM polymer side chains. Interestingly, the CP copolymer showed a strong inhibitory effect to Gram-positive and Gram-negative bacteria, promoted the proliferation of gingival fibroblasts, displayed significant hemostatic effects, and showed a low cytotoxic effect *in vitro*. Studies have shown that the inclusion of antibacterial, hemostatic, and proliferative properties are essential in a biomaterial for the treatment of oral ulcerative lesions. More importantly, the CP copolymer showed significant adhesive, anti-inflammatory, and hemostatic effects in the treatment of oral gingival ulcers in rats, indicating that this biodegradable, chitosan-based copolymer is a promising system for development as a therapeutic agent for oral ulcerative lesions.

## Data Availability Statement

All datasets generated for this study are included in the article/Supplementary Material.

## Ethics Statement

The animal study was reviewed and approved by the Animal Ethics Committee of Xiamen University.

## Author Contributions

YZ, LK, YL, QZ, GD, HW, and XZ conducted the experiments. YZ and LK analyzed the date. YZ, LK, and YL made the draft and finalized this manuscript. YZ and LK contributed equally to this work.

## Conflict of Interest

The authors declare that the research was conducted in the absence of any commercial or financial relationships that could be construed as a potential conflict of interest. The reviewer YW declared a shared affiliation, though no other collaboration, with one of the authors LK to the handling editor.
