# Supplementary material for: Enhanced Healing and Antimicrobial Efficacy of Chitosan-g-Polyacrylamide in a Rat Model of Gingival Ulcers
Source: Front Chem. 2020 Apr 24;8:273. doi: 10.3389/fchem.2020.00273 (PMC7193946; doi:10.3389/fchem.2020.00273)
Supplement: Supplementary file 1 [file Image_1.pdf]

Supplementary Materials

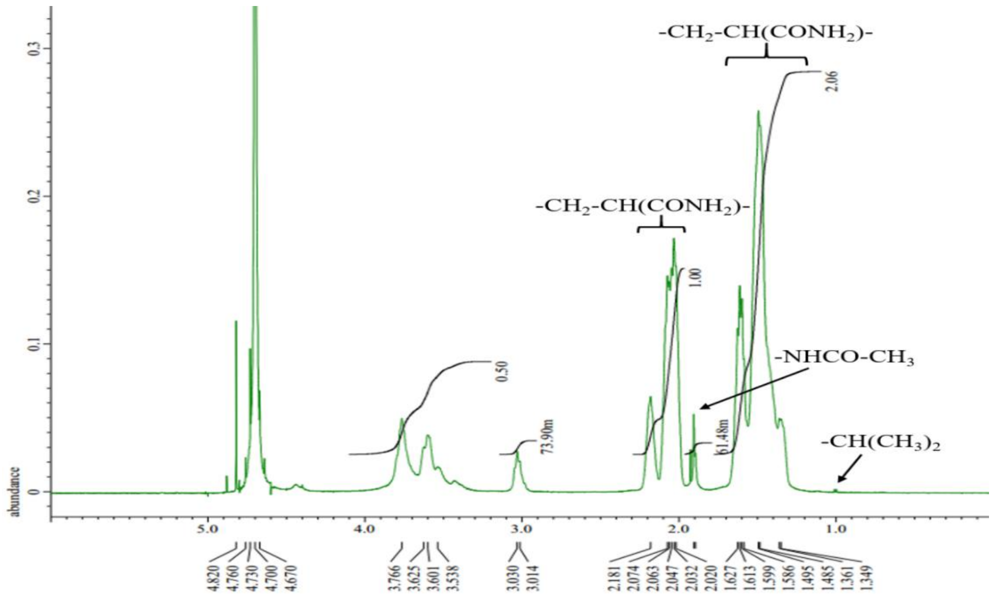

Supporting Figure S1. ^{1}H NMR spectra of PAM-CS copolymer, D_2O containing DCl 0.1 M as the solvent.

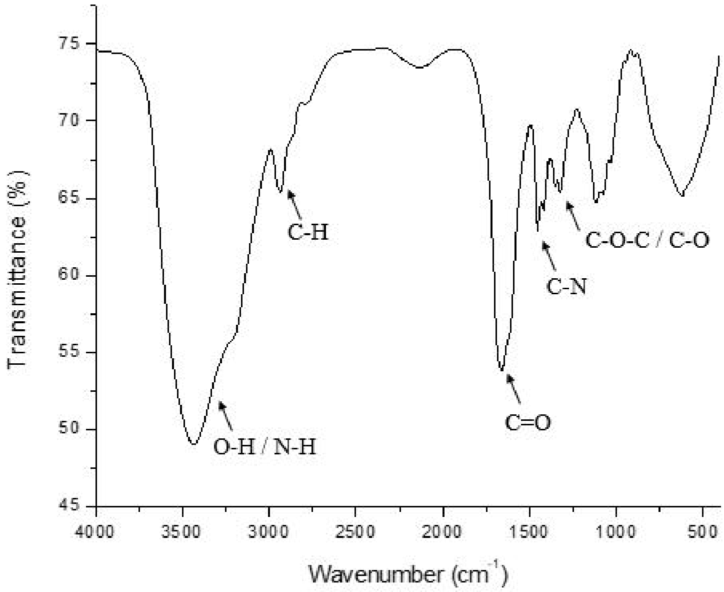

Supporting Figure S2. FTIR spectra of PAM-CS copolymer in potassium bromide pellet (4000-400cm^{-1} region) on Perkin-Elmer FTIR2000 spectrometer.
